# Perioperative Therapy of Oesophagogastric Adenocarcinoma: Mainstay and Future Directions

**DOI:** 10.1155/2017/5651903

**Published:** 2017-07-13

**Authors:** Katrin Bose, Caspar Franck, Meike N. Müller, Ali Canbay, Alexander Link, Marino Venerito

**Affiliations:** Universitätsklinik für Gastroenterologie, Hepatologie und Infektiologie, Otto-von-Guericke Universität, Leipziger Str. 44, 39120 Magdeburg, Germany

## Abstract

Perioperative chemotherapy improves overall survival in patients with oesophagogastric adenocarcinoma (OAC) and locoregional disease. The mainstay of perioperative chemotherapy in these patients is a platinum/fluoropyrimidine combination. The phase III FLOT4 trial has shown that the FLOT triplet regimen (oxaliplatin, infusional 5-FU, and docetaxel) improves the outcome of patients with OAC and locoregional disease as compared to the ECF triplet (epirubicin, cisplatin, and infusional 5-FU). Targeted therapies have currently no role in the perioperative setting for the treatment of patients with OAC. For patients with oligometastatic disease, upfront gastrectomy followed by chemotherapy did not show any survival benefit compared with chemotherapy alone and thus should be discouraged. Whether surgery should be offered to patients with metastatic OAC achieving a systemic control after upfront chemotherapy is under scrutiny in the phase III FLOT5/Renaissance trial. After neoadjuvant treatment, lymph node status but not pathologic tumor response is an independent factor in the prediction of overall survival. Growing evidence suggests that perioperative chemotherapy may be associated with an increased mortality risk in patients with microsatellite instable (MSI)/mismatch repair-deficient (MMRD) adenocarcinoma, thus validating poor responsiveness to chemotherapy in MSI patients with locoregional disease.

## 1. Oesophagogastric Adenocarcinoma: Epidemiological Aspects

Oesophagogastric adenocarcinoma (OAC) includes adenocarcinomas of the stomach (gastric cancer), of the distal oesophagus, and of the gastrooesophageal junction.

With 951,600 new cases globally recorded in 2012, gastric cancer is the fifth most common cancer worldwide, accounts for seven per cent of all new cases of cancer, and is the third most common cause of death from cancer [1, 2]. There is a significant variation in incidence worldwide. The highest rates are seen in less developed countries with about 35% in Eastern Asia. In Asian countries, because of the high incidence, screening for gastric cancer is a routine and the tumor is diagnosed in earlier stages than in western countries [3]. Symptoms include weight loss, dysphagia, dyspepsia, vomiting, early satiety, or iron deficiency anemia. Gastric cancer is commonly seen in patients over 50 years. In the United States, for example, the average age at diagnosis is 70 years [4]. Rates of gastric cancer are generally about twice high in men than in women. Five-year survival depends on the stage and differs among countries. For example, in Europe and US, it ranges from 25% to 63% if the cancer is diagnosed at an early stage [5]. Risk factors include male gender, tobacco use, high salt diet, processed meat, and low fruit and vegetable intake as well as obesity, atrophic gastritis, partial gastrectomy, Ménétrier's disease, genetic predisposition, and *Helicobacter pylori* infection [6–8]. A chronic infection with this bacterium is the strongest identified risk factor, with about 90% of new cases of gastric cancer worldwide [9–11].

Around 455,800 new cases of oesophageal cancer, thus 3% of all new cases of cancer, were recorded globally in 2012, of which 12% are adenocarcinomas [12]. The incidence rates of oesophageal cancer vary internationally by more than 21-fold with the highest rates in Eastern Asia and in Eastern and Southern Africa and the lowest rates in Western Africa [13]. Data about differences between the 2 main subtypes (adenocarcinoma and squamous cell carcinoma) are sparse and with high international variation. However, during the recent decades, the incidence and prevalence of adenocarcinoma of the gastrooesophageal junction were arising in developed countries whereas the incidence of gastric cancer is gradually declining [14]. Data about 5-year survival are heterogeneous as well. For example, in the United States, the five-year survival rate of oesophageal cancer is about 20%, and in Europe, it is about 10% [5]. The median age at diagnosis in the USA is 67 years [15].

With respect to the adenocarcinoma of the gastrooesophageal junction, incidence rates are usually three to four times higher in men than in women. The main known risk factors for adenocarcinoma of the gastrooesophageal junction are overweight, obesity, chronic gastrooesophageal reflux disease, Barrett's oesophagus, and achalasia as well as smoking and low intake of fruits and vegetables [16, 17].

## 2. Mainstay of Perioperative Chemotherapy in Patients with Oesophagogastric Adenocarcinoma

Since the results of the MAGIC trial have been published, perioperative chemotherapy has become the standard of care for patients with resectable OAC (74% had gastric and 12% had junctional cancer) [18]. In the study, patients were randomly assigned to 3 preoperative and postoperative cycles each of ECF (epirubicin, cisplatin, and 5-fluorouracil; *N* = 250) or surgery alone (*N* = 253). Patients receiving perioperative chemotherapy had a decreased tumor size and stage as well as significantly improved progression-free survival (PFS) and overall survival (OS) with an estimated improvement of 13 percentage points in the five-year survival rate.

Similar results were shown in the French multicenter phase III ACCORD trial. Overall, 224 patients (25% had gastric and 65% had junctional cancer) were randomized to receive surgery alone (*N* = 111) or combined with perioperative chemotherapy (1, 2, or 3 preoperative and 1 to 4 postoperative cycles consisting of 5-fluorouracil and cisplatin, *N* = 113). In line with the results of the MAGIC trial, the use of perioperative chemotherapy led to a 14% improvement in 5-year survival [19].

The need for a balance between intensity of neoadjuvant chemotherapy and acceptable toxicity represents a major challenge in the perioperative setting of patients with resectable OAC. In the multicenter, randomized phase III UK Medical Research Council OE 05 trial, patients with oesophageal and junctional adenocarcinoma received preoperatively either 2 cycles of cisplatin and 5-fluorouracil (*N* = 451) or 4 cycles of epirubicin, cisplatin, and capecitabine (*N* = 446) followed by oesophagectomy with 2-field lymphadenectomy. No significant differences were observed with respect to overall survival as well as postoperative complications and deaths at 30 and 90 days postsurgery. However, a significantly higher toxicity was observed in the arm with intensified chemotherapy. Thus, the addition of an anthracycline to a platinum/fluoropyrimidine-based chemotherapy is no longer justified and should be discouraged [3, 20, 21].

The addition of docetaxel to a platinum/fluoropyrimidine has been under scrutiny in the German multicenter, open-label, randomized phase 2/3 FLOT4 trial. The FLOT regimen consists of docetaxel, oxaliplatin, leucovorin, and 5-fluorouracil. This triplet with a very appealing name—FLOT in German (pronunciation *flott*) means quick or smart—is widely used in Germany in the palliative setting. Overall, in the FLOT4 trial, 714 patients with resectable OAC were randomly assigned to receive either 3 preoperative and 3 postoperative cycles of ECF/ECX (infusional 5-FU or oral capecitabine) or 4 preoperative and 4 postoperative cycles of FLOT. Results from the phase II part have been already published, showing a significantly higher proportion of patients achieving pathological complete regression in the FLOT group compared to the group receiving ECF/ECX (20 of 128 [16%; 95% CI 10–23] patients versus 8 of 137 [6%; 3–11] patients; *p* = 0.02) [22, 23]. Results of the phase III part of the trial have been presented at the ASCO 2017 meeting [24]. Overall, the wide majority of included patients had an ECOG of 0-1, whereas about 75% were younger than 70 years old. In this population, FLOT significantly increased rates of curative surgery and prolonged median PFS (18 versus 30 months) and median OS (35 versus 50 months) as compared to ECF/ECX. The relative effect of FLOT was consistent across subgroups and sensitivity analysis. Furthermore, there was no increase in surgical morbidity and mortality, resurgeries, or hospitalization time between the two groups. With respect to toxicity grades 3-4, patients receiving ECX/ECF had significantly more vomiting, nausea, thromboembolic events, and anemia, whereas patients on FLOT had more diarrhea, infections, neutropenia, and sensory neuropathy. Moreover, no differences were observed with respect to serious adverse events or toxic deaths. Thus, toxicity was drug-specific but overall similar between both regimens. Accordingly, FLOT can be considered the new standard of care in the perioperative treatment of OAC patients with a good performance status. In particular, as both FLOT and ECF are platinum/fluoropyrimidine-based triplets and taking into account the data from the OE 05 trial (4 cycles of neoadjuvant epirubicin, cisplatin, and capecitabine compared to 2 cycles of neoadjuvant cisplatin and capecitabine did not improve overall survival but delivered significantly higher toxicity), the advantage of FLOT over ECF/ECX appears to be mainly based on the higher effectiveness of docetaxel compared to epirubicin.

According to ESMO Guidelines, “perioperative (pre- and postoperative) chemotherapy with a platinum/fluoropyrimidine combination is recommended for patients with ≥Stage IB resectable gastric cancer” (i.e., OAC and locoregional disease in the absence of distant metastasis). It may be reasonable to use any platinum/fluoropyrimidine doublet or triplet before surgery. The duration should be 2-3 months each for the neoadjuvant and for the adjuvant part [3, 25]. Both platinum compounds cisplatin and oxaliplatin are effective, and the same is true for infusional 5-FU and oral capecitabine [26]. Thus, the choice of the compound to use should be only addressed according to the side effect profile of the cytostatic agents. The novel fluoropyrimidine S-1 containing tegafur (an inactive 5-FU prodrug) and the two enzyme inhibitors gimeracil and oteracil is as effective as infusional 5-FU with an improved safety profile [27, 28]. S-1 is licensed in advanced gastric cancer in combination with cisplatin only. Due to pharmacogenomics differences in western patients, the maximum tolerated dose of S-1 with cisplatin is lower than that in Asian patients. Data on perioperative S-1 are at the moment limited to Asian patients [29].

## 3. Does Targeted Therapy Play a Role in the Perioperative Setting?

According to the currently available evidence, targeted therapy is not a therapeutic standard in the perioperative setting of patients with OAC. However, several phase II/III trials are evaluating whether targeted therapies, which have led to encouraging improvement in the palliative treatment of OAC, may also add some benefit in the perioperative setting. For HER2-overexpressing OAC, the ongoing FLOT6 trial investigates the impact of the addition of trastuzumab and pertuzumab to perioperative FLOT on pathological response and survival [30]. Trastuzumab is a monoclonal antibody primarily developed for HER2-positive breast cancer. In the ToGA trial, the addition of trastuzumab to backbone chemotherapy led to a significant improvement of overall survival of patients with HER2-overexpressing OAC [31]. Pertuzumab is a HER2 dimerization inhibitor that prevents the pairing of HER2 with other HER receptors and may improve HER2 inhibition in combination with trastuzumab. An ongoing phase II study is comparing neoadjuvant cisplatin/fluoropyrimidine and trastuzumab alone or in combination with pertuzumab [32].

The UK Medical Research Council ST03 trial failed to provide any evidence for the use of bevacizumab, a monoclonal antibody against the vascular endothelial growth factor receptor (VEGFR), in combination with perioperative ECX for patients with resectable OAC. Furthermore, bevacizumab was associated with impaired wound healing [33].

Ramucirumab is a monoclonal antibody targeting VEGFR-2 that is recommended as monotherapy or in combination with paclitaxel in the palliative second-line treatment of OAC patients. For HER2-negative cancers, the currently recruiting FLOT7 (RAMSES) trial will address the value of ramucirumab in addition to perioperative FLOT [34].

Further phase II trials are currently investigating the addition of the PD-1 inhibitor pembrolizumab to chemotherapy in a perioperative setting [35, 36].

In conclusion, by today outside of studies, there is no evidence for targeted therapies in the perioperative treatment of OAC. However, several promising studies are currently recruiting and the first results may be expected soon.

## 4. Perioperative Therapy and Surgery for Oligometastatic Disease

Chemotherapy is the standard of care for incurable advanced OAC. Removal of the primary tumor from patients with metastatic disease confers a survival benefit in selected cancer types [37, 38]. However, whether additional surgery confers a survival benefit over chemotherapy alone in patients with OAC and oligometastatic disease remains controversial. At least partially, this question has been addressed in an open-label, randomized phase 3 trial at 44 centers or hospitals in Japan, South Korea, and Singapore [39]. Patients with advanced gastric cancer with a single noncurable factor confined to either the liver, peritoneum, or para-aortic lymph nodes were randomized (1 : 1) to receive gastrectomy followed by chemotherapy or chemotherapy alone. Chemotherapy consisted of oral S-1 80 mg/m^2^ per day on days 1–21 and cisplatin 60 mg/m^2^ on day 8 of every 5-week cycle. Gastrectomy was restricted to D1 lymphadenectomy without any resection of metastatic lesions. The primary endpoint was overall survival, analyzed by an intention to treat. Between February 2008 and September 2013, 175 patients were randomly assigned to chemotherapy alone (86 patients) or gastrectomy followed by chemotherapy (89 patients). After the first interim analysis, the predictive probability that the gastrectomy plus chemotherapy group would have a higher overall survival compared to the chemotherapy-alone group at the final analysis was only 13.2%. Therefore, the study was closed on the basis of futility. Overall survival at 2 years for all randomly assigned patients was 31.7% for patients assigned to chemotherapy alone compared with 25.1% for those assigned to gastrectomy plus chemotherapy. Median overall survival was 16.6 months for patients assigned to chemotherapy alone and 14.3 months for those assigned to gastrectomy plus chemotherapy. Based on the fact that gastrectomy followed by chemotherapy did not show any survival benefit compared with chemotherapy alone, the authors concluded that gastrectomy cannot be justified for the treatment of patients with advanced gastric cancer with a single noncurable factor. It should be noted that during this trial, five patients initially assigned to chemotherapy alone underwent gastrectomy with a curative intent because of complete disappearance of all noncurable factors during chemotherapy. This finding raises the question as to whether surgery should be offered to patients with metastatic OAC achieving a systemic control after upfront chemotherapy. The phase III FLOT5/Renaissance trial from the German AIO group will explore the effect of chemotherapy alone versus chemotherapy followed by surgical resection on survival and quality of life in patients with limited metastatic OAC and possibly answer this question [40]. The rationale for this study was provided by the previous phase II FLOT3-AIO trial in which 252 patients with resectable or metastatic OAC were enrolled [41]. Of the 238 eligible patients, 60 were classified as having limited metastatic stage and 36 of these 60 patients had surgery, including resection of the primary tumor and metastases. The median overall survival was significantly higher for patients who underwent surgery compared to the other patients (31.3 versus 15.9 months, resp.).

## 5. Prediction of Treatment Response

Despite the advances that were made in the treatment of resectable OAC, the identification of patients susceptible to relapse events remains challenging. In order to detect a prognostic marker, pathologic tumor response and lymph node status of 330 patients that underwent perioperative chemotherapy in the MAGIC trial were analyzed [42]. Pathologic response was scored on the basis of the Mandard tumor regression grading (TRG) system [43]. TRG 1 was referred to as complete regression in the resection samples, while TRG 5 described a tumor lacking signs of regression after the treatment. While pathologic tumor regression (TRG 1 or 2) was associated with improved survival in univariate analysis, multivariate analysis revealed lymph node metastases to be the only independent factor in the prediction of overall survival (HR 3.36, 95% CI 1.70 to 6.63). In line with former studies, these findings emphasize the prognostic role of lymph node status in contrast to tumor regression [44].

With respect to the microsatellite instability (MSI) status, the benefit of chemotherapy for patients with OAC in UICC stages II and III has been evaluated retrospectively in a large cohort of patients (*n* = 1276) with and without MSI [45]. Patients with stage II/III MSI-high OAC had a better prognosis when treated by surgery alone (hazard ratio 0.49, 95% CI 0.26–0.94) whereas the prognostic benefit of the MSI-high group compared to the MSI-low/MSS group was attenuated in patients receiving chemotherapy.

Recently, these results were confirmed in a secondary post hoc analysis of the MAGIC trial. MSI status was available for 303 patients and 20 of them had MSI. When treated with surgery alone, no significant difference in overall survival could be shown between MSS and MSI/mismatch repair-deficient (MMRD) tumor patients. Interestingly, perioperative chemotherapy even led to increased mortality risk in MSI patients (HR 2.18, 95% CI 1.08–4.42), thus validating poor responsiveness to chemotherapy in MSI patients [46].

## 6. Summary

The mainstay of perioperative chemotherapy in patients with OAC is a platinum/fluoropyrimidine-based doublet or triplet. FLOT is a triplet regimen which has been shown to be more effective than ECF/ECX and represents the new standard of care in OAC patients with good performance status undergoing perioperative chemotherapy. Ongoing studies are exploring the role of targeted therapies and of combined perioperative cytostatic therapy with surgery in the oligometastatic setting. Pathologic tumor regression is a marker for tumor response whereas lymph node metastases predict overall survival. Whether the adjuvant part of the perioperative chemotherapy is always worth striving for has not been answered yet. In particular, patients with missing pathologic tumor regression may not benefit from adjuvant chemotherapy. Furthermore, the role of postoperative chemotherapy in patients with postoperative negative nodal status has still to be addressed.
